# Trends in contraceptive use and distribution of births with demographic risk factors in Ethiopia: a sub-national analysis

**DOI:** 10.3402/gha.v8.29720

**Published:** 2015-11-09

**Authors:** Solomon Shiferaw, Muna Abdullah, Yared Mekonnen, Abdoulaye Maïga, Akanni Akinyemi, Agbessi Amouzou, Howard Friedman, Aluisio J. D. Barros, Sennen Hounton

**Affiliations:** 1 Department of Reproductive Health and Management, School of Public Health, Addis Ababa University, Addis Ababa, Ethiopia; 2 UNFPA – East and Southern Africa Regional Office, Johannesburg, South Africa; 3 Mela Research PLC, Addis Ababa, Ethiopia; 4 Institut Supérieur des Sciences de la Population, Ouagadougou University, Ouagadougou, Burkina Faso; 5 Department of Demography and Social Statistics, Obafemi Awolowo University, Ile Ife, Nigeria; 6 UNICEF, New York, NY, USA; 7 United Nations Population Fund (UNFPA), New York, NY, USA; 8 Federal University of Pelotas, Capão do Leão, Brazil

**Keywords:** Ethiopia, distribution of birth risks, modern contraceptive use, total fertility

## Abstract

**Background:**

Evidence shows that family planning contributes to the decline in child mortality by decreasing the proportions of births that are considered high risk. The main objective of the present analysis was to examine the trends in use of modern contraceptives and their relationship with total fertility rate (TFR) and distribution of births by demographic risk factors as defined by mother's age, birth interval, and birth order at the sub-national level in Ethiopia.

**Design:**

Analyses used data from three Demographic and Health Surveys in Ethiopia (2000, 2005, and 2011), which are nationally representative data collected through questionnaire-based interviews from women 15–49 using a stratified, two-stage cluster sampling. First, we examined the trends of and relationship between TFR (in the 3 years before each survey) and modern contraceptive use among currently married women in all administrative regions over the time period 2000–2011 using linear regression analysis. We also examined the relationship between birth risks and under-five mortality using the no-risk group as a reference. Finally, multiple logistic regression analysis was performed to estimate the relationship between the effect of being a resident in one of the regions and having an avoidable birth risk (which includes births to mothers younger than 18 and older than 34 years, birth interval of less than 24 months and birth order higher than third) after adjusting for select covariates including wealth, educational status, residence, religion and exposure to family planning information.

**Results:**

Sub-national-level regression analysis showed an inverse relationship between modern contraceptive use among married women and the TFR, with an average decrease of TFR by one child per woman associated with a 13 percentage point increase in modern contraceptive use between 2000 and 2011. A high percentage of births in Ethiopia (62%) fall in one of the risk categories (excluding first births), with wide regional variation from 55% in Gambela to 72% in the Somali region. The multivariate analysis showed women living in the Somali, Afar and Benishangul-Gumuz regions had significantly higher odds of having avoidable birth risk compared to those in Addis Ababa after controlling for observed covariates. The trend analysis showed there was a significant drop in the proportion of births from women above 34 years between 2000 and 2011. There was no significant decline in births to women less than 18 years between 2000 and 2011.

**Conclusions:**

A majority of births in Ethiopia fall in one of the risk categories, with substantial region-to-region variation in the percentage of births with avoidable risk factors, Somali and Afar having the highest burden. The analysis indicated that births in the three regions had significantly higher odds of having one of the avoidable risk factors compared to Addis Ababa, and we suggest family planning programmes need to identify differentials of modern contraceptive use at the sub-national level in order to better address coverage and equity issues.

**Paper context**

Family planning contributes to the decline in child mortality by decreasing the proportions of births that are considered high risk. A majority of births in Ethiopia (62%) fall in one of the risk categories, with substantial region-to-region variation. Births in Somali, Afar, and Benishangul-Gumuz regions had significantly higher odds of having one of the avoidable risk factors compared to Addis Ababa. Family planning programmes need to identify differentials of modern contraceptive use at the sub-national level in order to better address coverage and equity issues.

Family planning programmes contribute to improving child survival by reducing the proportion of ‘high-risk’ births (1–5). Generally, the three demographic groups of children who are considered to be at a higher risk of mortality are those born to very young mothers (less than 18 years of age), those born to older women (past 40 years of age), and those born too soon after a previous birth (less than 2 years) (2).

Evidence shows that there is a strong negative correlation between levels of contraceptive use and levels of high-risk births and infant mortality. High-risk births account for large proportions of births in developing regions. For example, narrowly spaced births and births to the youngest and oldest mothers account for up to 25 and 36% of births, respectively, in many sub-Saharan countries. Similarly, births to adolescents account for at least 15% of births in sub-Saharan African and Latin American countries (5). Like many low-income countries, Ethiopia has a high adolescent birth rate at 79 per 1,000, with more than one-third (34%) of women 20–49 years having given birth by age 18 and 20% of births occurring within 24 months of the previous birth (6).

With an average annual increase of modern contraceptive prevalence rate among married women (mCPR) of nearly 2 percentage points since 2000, the country has registered significant progress (6). Modern contraceptive use among currently married women nearly quadrupled from 6.3% in 2000 to 27.3% in 2011.

However, with an unmet need of 25% and demand satisfied only for 53% of women in 2011, there is substantial unfulfilled demand for modern contraceptives. In addition, the data show that progress is unevenly distributed. For example, the mCPR in the Somali region (which is the region of Ethiopia bordering Somalia) in 2011 was 3.8%, a figure well below the national average 11 years earlier. The capital city, Addis Ababa, has the highest mCPR among married women at 56%, followed by Gambela and Amhara regions at 33% each. Unmet need is highest in Oromia at 30% and lowest in Addis Ababa at 11%. With unmet need of 16% and met need of 10%, Afar has the lowest demand at 26%, compared to Addis Ababa, which has a demand of 73% (6).

The same region-to-region variation exists with regard to total fertility rate (TFR), which is the average number of children that would be born per woman if all women lived to the end of their childbearing years and gave birth to children according to a given fertility rate at each age (7). The TFR in 2011 ranged from 1.5 in Addis Ababa (below replacement level) and 4.0 in the Gambela region to 7.1 in the Somali region, while the national average was 4.8. Indeed, the Somali region saw an increase in TFR between 2000 (5.7) and 2011 (7.1) (6).

The rapid increase in access to and use of contraceptives was also associated with decreasing infant and under-five mortality rates, partly attributable to the launch of the health extension programme, which has deployed more than 30,000 government-salaried health extension workers in every sub-district (or *kebele*) of the country (8–10).

The aim of the present analysis is to examine 1) the trends in the use of modern contraceptives and their relationship with TFR, 2) distribution of birth risk, 3) trends in birth risk categories across regions, and 4) the relationship between the distribution of birth risk and under-five mortality in Ethiopia.

By showing the relative contribution of contextual factors (mainly residence in one of the regions) in reducing avoidable birth risks, the analysis will generate crucial information that will be useful to advocate for prioritisation of family planning programmes, particularly in regions with performance below the national average.

## Methods

### Design and study period

The analysis uses data from three Demographic and Health Surveys (DHS) in Ethiopia (2000, 2005, and 2011), which are repeat cross-sectional surveys by design. DHS collects questionnaire interview-based nationally representative data from women 15–49 using a stratified, two-stage cluster sampling that allows for specific indicators, such as modern contraceptive use, to be collected. By design, the survey allows the estimation of regional-level indicators. Questions asked include, among others, background characteristics such as age and education, as well as information about key indicators of maternal and child health including birth history, childhood mortality, and use of family planning methods. The surveys are carried out under the aegis of the Ministry of Health and are implemented by the Central Statistical Agency (CSA). ICF International provides technical assistance as well as funding to the project through the MEASURE DHS project, a project funded by the US Agency for International Development providing support and technical assistance in the implementation of population and health surveys in countries worldwide. The data are freely available from the CSA or the MEASURE DHS project (6, 11, 12).

First, we examined the trends of and relationship between TFR (in the 3 years before the survey) and mCPR (including method mix) in the 11 administrative regions over the time period 2000–2011 using linear regression analysis. We also examined the relationship between birth risks and under-five mortality by calculating the risk ratio with 95% confidence interval, using the ‘no-risk’ group (which is actually defined as births not associated with any specific risk) as a reference.

### Data processing

The main outcome of interest in this study was the percentage of births with avoidable birth risk for births in the 5 years preceding each survey. Birth risk categories were defined by three main variables: mother's age at birth, grouped into less than 18 years, 18–34 years, and 35–49 years; preceding birth interval, which is the length in months between previous birth and the index child, grouped into less than 18 months, 18–23 months, and 24 or more months; and birth order, grouped into first births, second or third births, and fourth or greater births. As described by Ross and Stover (2), birth risk categories include the following 11 mutually exclusive groups of children based on the percentage of children born in the 5 years preceding the survey. *Avoidable birth risk* refers to categories numbered 3 to 11 below (i.e. excluding first births in the optimal age range of 18–34, which is considered an unavoidable risk).

1. Not in any risk category
2. First birth to mother 18–34 years age
3. Mother's age <18 years
4. Mother's age >34 years
5. Birth interval <24 months
6. Birth order >3
7. Mother's age <18 years and birth interval <24 months
8. Mother's age >34 years and birth interval <24 months
9. Mother's age >34 years and birth order >3
10. Birth interval <24 months and birth order >3
11. Mother's age >34 years, birth interval <24 months and birth order >3

We used births in the preceding 5 years to have sufficient numbers for the analysis of births by all demographic risk factors (maternal age, inter-birth interval, and higher order births).

Independent variables include region (Addis Ababa, Afar, Amhara, Oromia, Somali, Benishangul-Gumuz, Southern Nations and Nationalities People [SNNP], Tigray, Harari, Gambela, and Dire Dawa), wealth quintile, educational status (no education, primary, secondary, higher) residence (urban, rural), and discussion about family planning in health facilities (yes, no).

Modern contraceptive use in this study refers to current use of modern family planning methods among currently married (or in union) women, which include pills, intrauterine devices, injectables, condoms, female sterilisation, vasectomy, implants, and the lactational amenorrhoea method.

Finally, multiple logistic regression analysis was performed to estimate the relationship between the effect of being a resident in one of the regions and having avoidable birth risk, after adjusting for select covariates including wealth, educational status, residence, and exposure to family planning information. Stata 12.1 was used for data management and analysis.

## Results

### Trends and regional variation in TFR and mCPR among married women and women in union

Overall, the Ethiopian DHS in 2000, 2005, and 2011 surveyed 15,367, 14,070, and 16,515 women aged 15–49 in 14,072, 13,721, and 16,702 households, respectively, with close to 70% of the sample coming from rural areas in all rounds. Distribution of the sample by region is shown in Table 1.

**Table 1:** Trends in TFR for births in the preceding 3 years by region and residence across time, Ethiopia DHS 2000 to 2011

Characteristic	TFR in 2000 (95% CI)	*n*	TFR in 2011 (95% CI)	*N*	Absolute change DHS 2000–2011
Total	5.5 (5.3, 5.7)	15,306	4.8 (4.5, 5.1)	16,438	−0.7
Residence					
Urban	3.0 (2.6, 3.4)	4,530	2.6 (−0.6, 5.9)	5,310	−0.4
Rural	6.0 (5.8, 6.3)	10,776	5.5 (5.1, 5.8)	11,128	−0.5
Region					
Addis Ababa^a^	1.8 (1.2, 2.5)	2,010	1.5 (0.9, 2.1)	1,738	−0.3
Dire Dawa^a^	3.5 (2.6, 4.3)	1,048	3.4 (2.6, 4.0)	1,086	−0.1
Harari	4.2 (3.1, 5.3)	906	3.8 (3.1, 4.6)	1,094	−0.4
Gambela	4.4 (2.3, 6.5)	873	4.0 (2.9, 5.0)	1,122	−0.4
Afar	4.4 (3.4, 5.4)	855	4.6 (4.1, 5.1)	1,283	0.2
Benishangul-Gumuz	5.0 (4.3, 5.8)	991	5.2 (4.3, 6.1)	1,257	0.2
Somali	5.1 (3.9, 6.3)	837	7.1 (5.9, 8.2)	913	2.0
Tigray	5.3 (4.7, 6.0)	1,300	5.0 (4.3, 5.6)	1,717	−0.4
Amhara	5.5 (5.0, 6.0)	1,903	4.2 (3.7, 4.6)	2,077	−1.4^b^
SNNP	5.6 (5.2, 5.9)	2,017	4.9 (4.3, 5.4)	2,027	−0.7
Oromia	6.1 (5.6, 6.5)	2,566	5.6 (4.9, 6.3)	2,124	−0.4

a City administration councils;

b statistically significant. *n*=number of women (un-weighted). TFR, total fertility rate; CI, confidence interval; DHS, Demographic and Health Survey; SNNP, Southern Nations and Nationalities People.

As shown in Table 1, the TFR in the 3 years preceding each survey declined significantly by 0.7 children per woman, from 5.5 (95% CI: 5.3, 5.7) to 4.8 (95% CI: 4.5, 5.1) between 2000 and 2011 at the national level. The region with the highest and statistically significant drop in TFR was Amhara (by 1.4 children per woman). TFR also showed relatively higher decline in Tigray (by 0.4 children per woman) and SNNP (by 0.7 child per woman), though not significantly different. On the other hand, the Somali region saw a reverse trend, with TFR increasing by two children per woman during the same period, although marginally significant (TFR in 2000=5.1, 95% CI: 3.9, 6.3 vs. TFR in 2011=7.1, 95% CI: 6.0, 8.2). There was no statistically significant change in TFR for the rest of the regions.

Modern contraceptive use among currently married women significantly increased by 21 percentage points, from 6.3 to 27.3% (*p*<0.05). As expected, the region with the highest drop in TFR (Amhara) also showed the highest increase in mCPR in 2000–2011, at 26.4 percentage points, while the Somali and Afar regions had the smallest increases in mCPR, at 1.4 and 1.7 percentage points, respectively, showing no statistically significant change over time. The changes in mCPR in Dire Dawa and Harari regions were not statistically significant, apparently because of small sample size, as detailed in Table 2.

**Table 2:** Trends in mCPR by region and residence across time, Ethiopia DHS 2000 to 2011

Characteristic	mCPR in 2000 (95% CI)	*n*	mCPR in 2011 (95% CI)	*n*	Absolute change DHS 2000–2011
Total	6.3 (5.2, 7.6)	9,380	27.3 (24.9, 29.9)	10,204	21.0^a^
Residence					
Urban	28.3 (24.4, 32.5)	1,843	49.5 (45.6, 53.3)	2,422	21.2^a^
Rural	3.3 (2.6, 4.1)	7,537	22.5 (20.1, 25.1)	7,782	19.2^a^
Region					
Addis Ababa^b^	34.3 (30.7, 38.1)	670	56.3 (52.4, 60.2)	634	22.0^a^
Dire Dawa^b^	23.5 (12.6, 27.7)	526	31.7 (24.6, 39.8)	626	8.2
Harari	19.0 (12.6, 27.7)	482	31.5 (25.4, 38.3)	635	12.5
Gambela	12.3 (7.8, 18.9)	656	33.2 (24.2, 43.6)	768	20.9^a^
Afar	7.4 (3.9, 13.6)	619	9.1 (5.4, 14.8)	960	1.7
Benishangul-Gumuz	8.5 (5.8, 12.3)	711	26.3 (19.9, 33.9)	904	17.8^a^
Somali	2.4 (0.5, 10.3)	569	3.8 (1.6, 8.6)	664	1.4
Tigray	9.3 (5.5, 15.3)	868	21.2 (17.1, 26.0)	984	11.9^a^
Amhara	6.6 (4.7, 9.3)	1,315	33.0 (28.6, 37.8)	1,331	26.4^a^
SNNP	5.0 (3.0, 8.3)	1,312	24.7 (20.1, 29.9)	1,295	19.7^a^
Oromia	4.3 (2.8, 6.6)	1,652	24.9 (20.6, 29.8)	1,403	20.6^a^

a Statistically significant;

b city administration councils. *n*=number of women (un-weighted). mCPR, modern contraceptive prevalence rate; CI, confidence interval; SNNP, Southern Nations and Nationalities People; DHS, Demographic and Health Survey.

### Trends in the distribution of method mix

In terms of method mix, the 3-month injectable (medroxyprogesterone acetate) is the most commonly used modern contraceptive method in Ethiopia, accounting for 72.6% of the method mix in 2011, followed by implants and pills at 12 and 7.4%, respectively. Trend analysis showed that implant use increased (from 1 to 12%) while pill use decreased (from 31 to 7.4%), as did traditional methods (from 1.7 to 1.3%). However, the pattern is slightly different in the Somali region, where pill use is as high as 20% of the method mix and injectables constituted 47% in 2011. Details are shown in Supplementary Table 1.

### Relationship between modern contraceptive use and TFR

**Fig. 1:**
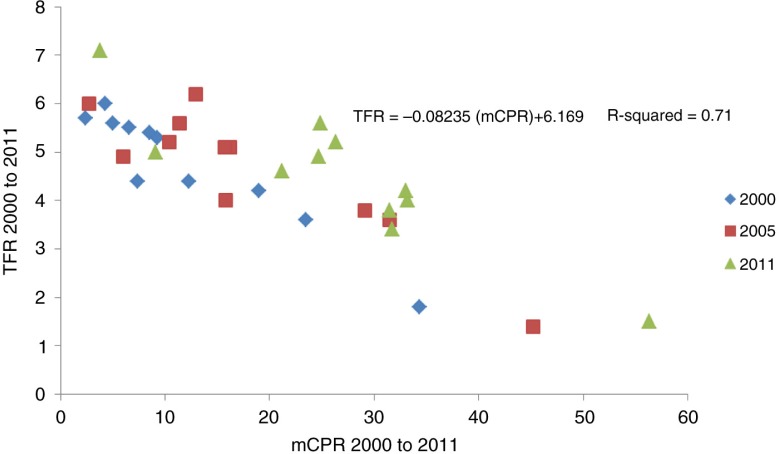
Relationship between changes in mCPR and TFR for all regions between 2000 and 2011.

As shown in Fig. 1, there was a significant negative linear relationship [TFR=−0.08235 (mCPR)+6.169; *p*<0.001] between the levels of modern contraceptive use among married women and TFR across the different regions of Ethiopia between 2000 and 2011. The analysis suggests that the TFR declined on average by one child per woman for every increase in the mCPR of about 13 percentage points. It also shows that the TFR would be around 6.2 if there were no modern contraceptive use.

### Levels and trends of birth risk categories for births in the preceding 5 years by region

**Fig. 2:**
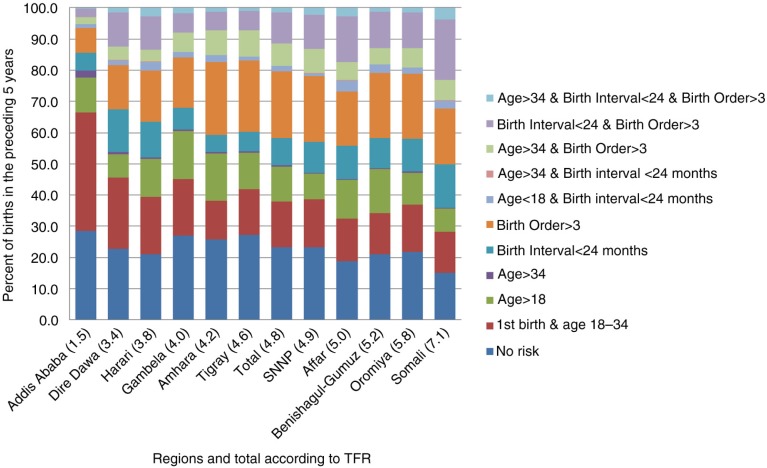
Distribution of births in the preceding 5 years by risk factor and TFR across regions, Ethiopia DHS2011.

As shown in Fig. 2, the proportion of births with avoidable risk (any risk factor other than first birth) was highest in Somali at 72% and Afar at 68%, followed by Benishangul-Gumuz (66%), Oromia (63%), and Amhara (62%). Addis Ababa and Dire Dawa (both cities with better access to health services) had lower percentages of births with any risk factors, at 34 and 54%, respectively. Interestingly, Gambela (a relatively underdeveloped region) also had a lower percentage of births with avoidable birth risk, at 55%.

**Fig. 3:**
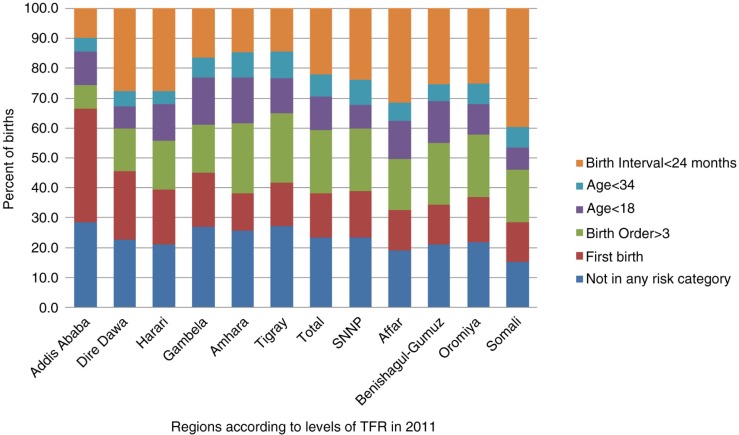
Distribution of births in the preceding 5 years by single risk factor, Ethiopia DHS 2011.

As Fig. 3 shows, the most common single risk factors in 2011 were narrowly spaced births (22%) and births to mothers older than 34 years of age (21%), followed by births to mothers younger than 18 years of age (11%). Birth risk associated with higher parity (birth order greater than three) was the least common, at 8%. The main single risk factor that was consistently higher in the two regions with higher fertility (Somali and Afar) compared to the national average was the proportion of births with short birth interval, at 40 and 32%, respectively.

Addis Ababa had the lowest percentage of births with short birth interval at 10%, followed by Tigray (15%) and Amhara (15%). As expected, Addis Ababa and Dire Dawa (both city administrations) also had higher proportions of first births, at 38 and 23%, respectively, compared to the national average of 15%.

Supplementary Table 2 shows that the proportion of births to mothers older than 34 years was the only single risk factor that showed a statistically significant drop between 2000 and 2011 at the national level, moving from 9.4% (95% CI: 8.7, 10.0) to 7.6% (95% CI: 7.1, 8.0). Looking at regional variations, there was a significant decline in the percentage of births with less than a 24-month birth interval in Addis Ababa, from 17.2% (95% CI: 14.8, 20.0) to 9.9% (95% CI: 7.3, 13.4), while the percentage of first births increased significantly from 26.5% (95% CI: 23.3, 30.1) to 37.9 (95% CI: 32.9, 43.2). It is important to note that the Gambela and Amhara regions had a significantly higher percentage of births to mothers younger than 18 years, at 15.6% (95% CI: 12.7, 19.0) and 15.2% (95% CI: 14.1, 16.3), respectively, compared to the national average of 11% (95% CI: 10.4, 11.8) in 2011.

### Levels and trends in distribution of births in the preceding 5 years with avoidable risk

As shown in Table 3, the Somali region had the highest proportion of avoidable birth risk (82.5%) in 2011, compared to the national average of 72.6%. While the changes were not statistically significant, the Somali and Afar regions showed a slight increase in the proportion of avoidable birth risk from 2000 to 2011.

**Table 3:** Trends in proportion of births in the preceding 5 years for which the birth risk is avoidable by region, Ethiopia DHS 2000 to 2011

	2000			2011		
		
Region	*n*	%	95% CI	*n*	%	95% CI
Tigray	1,365	68.8	(67.0, 70.7)	1,984	68.1	(66.1, 70.0)
Afar	192	74.2	(71.2, 77.1)	2,046	78.1	(75.9, 80.2)
Amhara	6,037	71.3	(69.7, 72.7)	2,443	70.6	(68.3, 72.8)
Oromia	8,761	75.3	(73.5, 77.0)	2,914	74.2	(72.3, 76.1)
Somali	288	81.6	(79.8, 83.3)	2,024	82.5	(80.9, 84.0)
Benishangul-Gumuz	204	73.4	(70.9, 75.8)	1,857	75.7	(73.1, 78.2)
SNNP	4,411	71.5	(69.8, 73.2)	2,868	72.4	(70.7, 73.9)
Gambela	40	66.5	(63.3, 69.6)	1,241	67.1	(64.3, 69.7)
Harari	42	78.3	(74.5, 81.6)	913	74.2	(69.9, 78.1)
Addis Ababa^a^	190	65.0	(61.6, 68.3)	240	54.2	(46.9, 60.8)^b^
Dire Dawa^a^	54	70.4	(66.7, 73.8)	903	71.8	(66.1, 74.6)
Total	22,000	72.8	(71.9, 73.7)	19,433	72.6	(71.5, 73.7)

a City administration councils;

b statistically significant. CI, confidence interval; SNNP, Southern Nations and Nationalities People; DHS, Demographic and Health Survey.

Our analysis did not show a significant decline in the distribution of avoidable birth risk distribution within regions, except in Addis Ababa, where it dropped from 70.9% (95% CI: 68.7, 73.1) in 2000 to 61.1% (95% CI: 57.6, 64.5) in 2011.

### Birth risk categories and under-five mortality

As shown in Table 4, among the different birth risk categories, the highest risk ratio for under-five mortality was for children who had the combined risk of short birth intervals and mothers under age 18 and/or over the age of 34, compared to the reference group of births with no risk factor, although there fewer cases in those categories. Among the single risk factors, births to mothers aged less than 18 (adolescent pregnancy, which often happens due to child marriage) and births with an interval of less than 24 months had higher odds of under-five mortality (risk ratio=1.77, 95% CI: 1.63, 1.92). The risk ratio represents the ratio of the proportion of births in the last 5 years in each risk category that had died to the proportion dead among births not in any risk category (reference category).

**Table 4:** Risk ratios for under-five mortality by distribution of birth risks in the 5 years preceding the survey, Ethiopia DHS 2011

Birth risk category	Number of births	Number of deaths	Under-five mortality	Risk ratio (95% CI)
Not in any risk category (reference)	37,738	388	76.9	1.0
Mother's age >34 and birth order >3	19,927	235	86.4	1.11 (1.01, 1.22)*
Birth order >3	40,918	482	87.7	1.13 (1.02, 1.23)*
First birth to mothers age 18–34	22,448	275	88.7	1.14 (1.04, 1.25)*
Mother's age >34	961	17	118.0	1.48 (1.36, 1.61)*
Birth interval <24 months and birth order >3	18,133	500	197.3	2.30 (2.13, 2.49)*
Mother's age <18	11,454	223	146.7	1.77 (1.63, 1.92)*
Birth interval <24	12,970	257	144.7	1.77 (1.63, 1.92)*
Mother's age >34 and birth interval <24 months and birth order >3	5,122	121	170.2	2.04 (1.88, 2.20)*
Mother's age <18 and birth interval <24 months	1,606	45	211.9	2.45 (2.26, 2.65)*
Mother's age >34 and birth interval <24 months	65	3	325.0	3.43 (3.19, 3.70)*

### Multivariate analysis: determinants of avoidable risk births

Table 5 presents the relationship between avoidable birth risk and region after adjusting for selected covariates including wealth quintile, educational status (no education, primary, secondary, higher), residence (urban, rural), discussion about family planning in health facilities (yes, no), and religion (Christian, Muslim, traditional/other).

**Table 5:** Results from multiple logistic regression analysis of predictors of avoidable birth risk for births in the preceding 5 years, Ethiopia DHS 2011

		95% CI	
		
Characteristics	AOR	Lower limit	Upper limit
Region (Addis Ababa^a^ – ref)			
Afar	1.68	1.11	2.53
Amhara	1.00	0.69	1.44
Oromia	1.18	0.82	1.69
Somali	2.16	1.33	3.51
Benishangul-Gumuz	1.48	1.01	2.17
SNNP	1.18	0.82	1.70
Gambela	1.12	0.78	1.61
Harari	1.28	0.84	1.93
Tigray	0.97	0.67	1.40
Dire Dawa^a^	1.10	0.74	1.65
Women's education (no education – reference)			
Primary	0.84	0.71	1.00
Secondary	0.59	0.40	0.88
Tertiary	0.51	0.28	0.91
Told of family planning at health facility (*yes* – reference)			
No	1.18	1.03	1.35
Residence (urban – ref)			
Rural	1.54	1.19	1.98

a City administration councils. AOR, adjusted odds ratio (adjusted for wealth quintile and religion); CI, confidence interval; SNNP, Southern Nations and Nationalities People; DHS, Demographic and Health Survey.

As shown in Table 5, births in the Somali region (adjusted odds ratio [AOR]=2.16, 95% CI: 1.33, 3.51), Afar region (AOR=1.68, 95% CI: 1.11, 2.53), and Benishangul-Gumuz region (AOR=1.48, 95% CI: 1.01, 2.17) had significantly higher likelihoods of having avoidable risk compared to the reference region, Addis Ababa. Similarly, rural residence was significantly associated with avoidable birth risk (AOR=1.54; 95% CI: 1.19, 1.98). Women with any form of formal education (primary, secondary, or tertiary) had significantly lower odds of having avoidable risk, with the highest reduction for women with tertiary education (AOR=0.51, 95% CI: 0.28, 0.91). Women who reported not having had any discussion about family planning in health facilities were also significantly more likely to have avoidable birth risks (AOR=1.18, 95% CI: 1.03, 1.35). Wealth quintile and religion were not associated with avoidable birth risk.

## Discussion

Over the 11-year study period, a majority of births in Ethiopia fell into one of the risk categories, with substantial region-to-region variation, the Somali and Afar regions having the highest burden. The analysis indicated that births in the Somali, Afar, and Benishangul-Gumuz regions had significantly higher odds of having one of the avoidable risk factors compared to Addis Ababa. This is consistent with previous studies showing low contraceptive use in Somali and Afar regions (13–15).

The present analysis suggests that the TFR declined on average by one child per woman for every increase in the mCPR of about 13 percentage points, which is lower compared to developing countries as a whole (average decrease of TFR by one child per woman for every increase in the mCPR of 17 percentage points) (2). This difference may indicate that factors other than contraception (such as better access to education and health services) are playing important roles in bringing down fertility. The observed inverse relationship between changes in TFR and mCPR points to the fact that regions with low modern contraceptive use have higher fertility, which in turn is associated with higher prevalence of births with risk factors (2).

The majority of births in Ethiopia (62%) fall into one of the high-risk categories (excluding first births), compared to the average for developing countries of 56% (2). The most common single risk factor is short birth interval (less than 24 months), which is particularly true for the Somali and Afar regions, where nearly 4 and 3 out of 10 births, respectively, have birth intervals of less than 24 months.

The trend analysis showed there was a significant drop in the proportion of births to mothers older than 34 years between 2000 and 2011 at the national level, while there was a significant decrease in the percentage of births with short birth intervals in Addis Ababa. There was no significant decline in births to women younger than 18 years between 2000 and 2011, so this remains an important risk factor, particularly in regions such as Amhara and Gambela where child marriage is a significant problem (16). Our findings add to the evidence that preventing child marriage and offering adolescents the possibility to protect themselves from unplanned pregnancy could contribute to reducing high-risk births and under-five mortality.

On the other end of the spectrum are cities like Addis Ababa and Dire Dawa, where the proportions of first births are higher, at 30 and 21%, respectively. These figures are in contrast to the national average of 13%. Increasing proportion of first births is a global trend as countries transition from high to low fertility. Studies show the relative impact of contraceptive use in reducing child mortality arising from high parity decreases as countries transition to low fertility, partly because of the increase in the proportion of high-risk first births (2, 17).

Among the single risk factors, births to women younger than 18 years and short birth intervals were associated with higher odds of under-five mortality, which is consistent with previous studies in Ethiopia (18, 19) and elsewhere (17, 20, 21).

The analysis also revealed that the trend in method mix in regions with relatively low modern contraceptive use (e.g. the Somali region) has been characterised by an increase in the use of pills [which have a higher rate of discontinuation (6)], a decrease in the use of injectables, and almost non-existent use of long-term methods such as implants. Hence, family planning programmes need to prioritise provision of wider method choices, including those with better effectiveness and convenience.

The multivariate analysis indicated that women in the Somali, Afar, and Benishangul-Gumuz regions had significantly higher likelihood of having avoidable risk births compared to the reference region (Addis Ababa) regardless of residence, education, religion, wealth, or discussion about family planning in health facilities. Being a rural resident and having no education were significantly associated with higher likelihood of avoidable birth risk, which can be partly explained by the fertility preferences of families of similar background (22). For example, the mean ideal number of children for all women in the Somali and Afar regions was 9.7 and 7.4, respectively, in 2011, compared to the national average of 4.3 (6). These data indicate the need to target context-specific beliefs and traditions around fertility preference, as achieving the goal of meeting current demand may not result in significant fertility decline in the foreseeable future.

There are a few limitations to this study. First, fertility is affected by multiple factors including fertility preference and intention, which may not be affected directly by a mere improvement in access to contraceptives. Second, the cross-sectional nature of the surveys precludes making temporal associations between contraceptive use and birth risk distributions, as both are measured at the same time. Finally, although we have attempted to account for variations in important individual-level factors, we must be cautious in making inferences at the regional level as there is substantial diversity in socio-cultural practices and beliefs within regions.

Nevertheless, the consistency of the relationship between the levels of contraceptive use, fertility behaviour of women, and the regional distribution of births with demographic risk factors indicate the possibility of cofounding as an alternative explanation is unlikely.

## Conclusions

The analyses revealed that there was a significant negative correlation between the level of modern contraceptive use among married women and TFR across the different regions of Ethiopia between 2000 and 2011, suggesting that the TFR declined on average by one child per woman for every increase in the mCPR of about 13 percentage points.

A majority of births in Ethiopia (62%) fell into one of the risk categories (excluding first births). There was substantial variation in the percentage of births with avoidable risk factors, however, from 55% in Gambela to 72% in the Somali region and 70% in Afar. The analysis indicated that the Somali region also has a slightly different pattern of method mix in favour of short-acting methods (pills), which could limit impact.

The trend analysis showed there was a significant drop in the proportion of births to women older than 34 years between 2000 and 2011 at the national level, while there was a significant decrease in the percentage of births with short birth interval only in Addis Ababa. There was no significant decline in births to women younger than 18 years between 2000 and 2011, so this remains an important risk factor, particularly in regions such as Amhara and Gambela where there are high rates of child marriage.

Living in the Somali and Afar regions is significantly associated with having higher odds of avoidable birth risk after controlling for observed covariates including wealth status, residence, education, religion, and discussion about family planning in health facilities.

Although Ethiopia has made significant progress in improving modern contraceptive uptake and decreasing high fertility, substantial regional variations persist, pointing the need to refocus family planning programmes to promote more equitable access to a broader choice of effective contraceptive methods throughout the country. Effort should also be made to prevent child marriage, which is often a precursor for teenage pregnancy and high-risk births.

## Supplementary Material

Trends in contraceptive use and distribution of births with demographic risk factors in Ethiopia: a sub-national analysis
