# Supplementary material for: Trends in contraceptive use and distribution of births with demographic risk factors in Ethiopia: a sub-national analysis
Source: Glob Health Action. 2015 Nov 9;8:10.3402/gha.v8.29720. doi: 10.3402/gha.v8.29720 (PMC4642368; doi:10.3402/gha.v8.29720)
Supplement: Trends in contraceptive use and distribution of births with demographic risk factors in Ethiopia: a sub-national analysis [file GHA-8-29720-s001.pdf]

Supplementary File for Shiferaw et al. Trends in contraceptive use and distribution of births with demographic risk factors in Ethiopia: a sub-national analysis. Global Health Action 2015; 8:29720

Supplementary Table 1: Trends in distribution of currently used contraceptive method among currently married women age 15-49

**2011**										
**Region**	**Pill**	**IUD**	**Injection**	**Condom**	**Female sterilisation**	**Periodic abstinence**	**Withdrawal**	**Other**	**Implants**	**LAM**
Tigray	9.3	0.0	57.6	2.2	1.2	3.7	0.4	0.4	25.2	0.0
Affar	13.2	0.0	80.1	0.0	0.0	2.0	2.0	0.8	2.1	0.0
Amhara	4.4	0.9	78.2	0.0	1.8	1.4	1.0	0.4	11.7	0.2
Oromiya	8.3	1.0	71.7	0.4	0.8	4.0	0.9	0.0	12.8	0.0
Somali	19.5	0.0	47.3	9.7	0.0	11.3	0.0	0.0	12.2	0.0
Benishangul-Gumuz	10.1	0.0	78.4	1.0	2.2	2.3	0.4	0.0	5.6	0.0
SNNP	5.3	1.2	75.6	0.4	2.1	2.5	1.5	0.3	11.1	0.0
Gambela	13.0	1.9	78.2	2.5	1.4	1.8	0.0	0.0	1.3	0.0
Harari	19.3	3.4	55.3	2.7	0.9	8.4	1.0	0.0	8.5	0.4
Addis	17.5	4.1	57.2	3.1	3.7	8.1	1.5	0.2	4.6	0.0
Dire Dawa	13.8	3.4	45.2	6.2	0.7	6.0	0.4	0.0	23.6	0.0
**Total**	**7.4**	**1.2**	**72.6**	**0.6**	**1.6**	**3.2**	**1.0**	**0.2**	**12.0**	**0.1**
**2005**										
**Region**	**Pill**	**IUD**	**Injection**	**Condom**	**Female sterilisation**	**Periodic abstinence**	**Withdrawal**	**Other**	**Implants**	**LAM**
Tigray	17	0	80	1	0	2	0	1	0	0
Affar	20	0	68	3	0	9	0	0	0	0
Amhara	22	1	73	1	1	2	1	1	0	0
Oromiya	25	1	63	1	2	3	3	0	2	0
Somali	0	0	87	0	0	13	0	0	0	0
Benishangul-Gumuz	12	0	76	1	3	6	1	0	0	1
SNNP	16	0	75	1	0	2	1	3	1	0
Gambela	16	0	81	3	0	1	0	0	0	0

Harari	17	5	60	2	0	13	1	0	2	0
Addis	19	7	41	4	3	16	4	4	2	0
Dire Dawa	20	2	63	4	1	7	1	3	0	0
**Total**	**21**	**1**	**67**	**1**	**1**	**4**	**2**	**1**	**1**	**0**
**2000**										
**Region**	**Pill**	**IUD**	**Injection**	**Condom**	**Female sterilisation**	**Periodic abstinence**	**Withdrawal**	**Other**	**Implants**	**LAM**
Tigray	20	0	63	7	1	7	1	0	0	NA
Affar	56	0	31	0	9	4	0	0	0	NA
Amhara	40	0	45	0	3	9	0	2	1	NA
Oromiya	29	1	24	6	5	31	4	0	0	NA
Somali	49	2	14	18	12	5	0	0	0	NA
Benishangul-Gumuz	25	0	65	0	7	0	2	1	0	NA
SNNP	28	0	48	1	2	17	3	1	0	NA
Gambela	57	0	31	0	3	9	0	0	0	NA
Harari	37	3	33	8	1	8	2	3	5	NA
Addis	28	8	28	3	7	18	3	2	2	NA
Dire Dawa	31	7	36	4	1	15	1	1	4	NA
**Total**	**31**	**1**	**38**	**3**	**4**	**18**	**2**	**1**	**1**	NA

Supplementary Table 2: Trends in percentage of risk categories for births in the preceding 5 years between 2000 and 2011 by region

Region (TFR)	Year of survey	No risk category % (95%CI)	First birth % (95%CI)	BO>3 % (95%CI)	Age<18 % (95%CI)	Age>34 % (95%CI)	BI<24 months % (95%CI)
Addis Ababa (1.5)	2000	25.7 (23.9,27.6)	26.5 (23.3,30.1)	13.4 (11.3,15.7)	11.8 (9.9,13.9)	5.4 (4.3,6.9)	17.2 (14.8,20.0)
	2011	28.6 (25.5,31.8)	37.9 (32.9,43.2)	7.9 (5.5,11.3)	11.1 (8.5,14.2)	4.5 (3.2,6.3)	9.9 (7.3,13.4)
Dire Dawa (3.4)	2000	23.3 (20.9,25.8)	21.4 (18.5,24.6)	15.9 (13.7,18.4)	6.1 (4.5,8.2)	7.2 (5.1, 10.0)	26.1 (22.8,29.7)
	2011	22.7 (20.2,25.5)	22.9 (19.9,26.2)	14.2 (11.9,16.9)	7.4 (6.1,9.1)	5.0 (3.8,6.7)	27.7 (24.2,31.5)
Harari (3.8)	2000	18.2 (15.9,20.7)	16.3 (13.1,20.0))	18.3 (15.5,21.6)	10.4 (8.7,12.2)	6.9 (5.8,8.2)	29.9 (26.5,33.7)
	2011	21.08 (18.6,23.8)	18.23 (14.7,22.4)	16.4 (14.2,18.8)	12.3 (10.3,14.6)	4.4 (3.4,5.7)	27.6 (23.7,31.9)
Gambela (4.0)	2000	28.0 (25.8,30.2)	16.5 (13.9,19.4)	20.5 (17.8,23.6)	15.4 (12.6,18.7)	6.0 (4.7,7.9)	13.6 (10.9,16.6)
	2011	26.9 (25.0,28.9)	18.1 (15.3,21.3)	16.1 (13.6,19.0)	15.6 (12.7,19.0)	6.8 (5.4,8.5)	16.4 (14.1,19.1)
Amhara (4.2)	2000	25.6 (24.4,26.8)	11.1 (10.2,12.0)	23.1 (21.9,24.2)	14.4 (13.2,15.7)	9.5 (8.3,10.9)	16.4 (14.8,18.0)
	2011	25.7 (24.1,27.5)	12.5 (11.3,13.8)	23.3 (21.7,24.9)	15.2 (14.1,16.3)	8.4 (7.5,9.4)	14.8 (13.1,16.7)
Tigray (4.6)	2000	27.1 (25.7,28.5)	13.1 (11.9,14.3)	23.3 (21.6,25.0)	11.5 (10.5,12.6)	10.3 (9.1,11.8)	14.8 (13.6,16.0))
	2011	27.3 (25.8,28.8)	14.6 (13.1,16.1)	23.0 (21.4,24.6)	11.9 (10.5,13.5)	8.8 (7.5,10.3)	14.5 (12.9,16.3)
SNNP (4.9)	2000	23.7 (22.5,25.0)	16.7 (15.4,18.0)	20.3 (19.2,21.3)	6.8 (5.9,7.8)	10.7 (9.7,11.9)	21.8 (20.2,23.5)
	2011	23.4 (22.2,24.6)	15.4 (14.2,16.8)	20.9 (19.8,22.1)	8.0 (7.0,9.2)	8.2 (7.2,9.3)	24.0 (22.1,26.0)
Affar (5.0)	2000	21.1 (18.9,23.3)	18.3 (16.11,20.6)	16.8 (15.0,18.7)	9.6 (7.8,11.9)	7.9 (6.5,9.6)	26.4 (23.6,29.5)
	2011	18.9 (17.4,20.6)	13.5 (11.6,15.6)	17.2 (15.6,19.0)	12.5 (11.3,13.8)	6.3 (5.1,7.8)	31.5 (28.9,34.2)
Benishangul-	2000	22.7 (20.9,24.7)	14.5 (12.6,16.7)	20.6 (18.5,22.9)	12.1 (10.5,14.0)	7.5 (6.3,9.0)	22.5 (19.8,25.4)
Gumuz(5.2)	2011	21.1 (19.0,23.3)	13.2 (11.9,14.7)	20.8 (19.3,22.3)	14.0 (12.5,15.6)	5.6 (4.5,6.9)	25.4 (22.6,28.3)
Oromiya (5.8)	2000	21.2 (19.9,22.6)	14.0 (13.1,15.0)	22.4 (21.3,23.4)	9.5 (8.7,10.5)	8.7 (7.8,9.6)	24.2 (22.5,26.0)
	2011	21.9 (20.5,23.4)	15.0 (13.8,16.3)	20.9 (19.7,22.0)	10.3 (9.2,11.4)	6.7 (5.9,7.6)	25.2 (23.2,27.4)
Somali (7.1)	2000	16.0 (14.6,17.5)	13.2 (10.4,16.7)	19.8 (17.2,22.8)	8.3 (7.5,9.1)	6.1 (5.1,7.3)	36.6 (34.2,39.2)
	2011	15.2 (14.0,16.5)	13.1 (11.9,14.5)	17.7 (16.3,19.1)	7.5 (6.0,9.2)	6.8 (5.7,8.1)	39.7 (37.2,42.3)
Total (4.8)	2000	23.4 (22.6,24.1)	13.9 (13.3,14.5)	21.9 (21.3,22.6)	10.5 (9.9,11.1)	9.4 (8.7, 10.0)	21.0 (19.9, 22.0)
	2011	23.4 (22.6,24.2)	14.7 (14.0,15.4)	21.3 (20.6,22.0)	11.1 (10.4,11.8)	7.6 (7.1,8.0)	22.1 (20.8,23.3)
